# Effect of Surface Finishing and Nitriding on the Wetting Properties of Hot Forging Tools

**DOI:** 10.3390/ma18010172

**Published:** 2025-01-03

**Authors:** Jan Kapuściński, Łukasz Macyszyn, Michał Zielinski, Artur Meller, Michał Lehmann, Tomasz Bartkowiak

**Affiliations:** 1 Faculty of Mechanical Engineering, Institute of Mechanical Technology, Poznan University of Technology, Piotrowo 3, 60-695 Poznan, Poland; jan.kapuscinski@student.put.poznan.pl (J.K.); lukasz.macyszyn@put.poznan.pl (Ł.M.); tomasz.bartkowiak@put.poznan.pl (T.B.); 2 Fabryka Armatur Swarzędz, Świerkowa 27, 62-020 Rabowice, Poland; artur.meller@fa-swarzedz.com.pl (A.M.); michal.lehmann@fa-swarzedz.com.pl (M.L.)

**Keywords:** hot forging, wetting, lubrication, surface finish, surface metrology

## Abstract

Lubrication is a critical aspect of the metal forming process and it is strongly influenced by the surface texture of the tool-forming surfaces. This study is focused on determining the effect of surface finish and heat treatment on wettability involving commonly used lubrication agents. Three different finishing states are evaluated (as-ground, as-polished and as-nitrided). Surface topography was measured using a focus variation microscope. Parametric evaluation was carried out according to ISO 25178, including fractal methods. The functional relations between the finish state and wettability, lubricating agent and wettability, selected surface parameters and wettability, as well as between finish state and selected surface parameters, were designated. The results showed that surface finishing treatments, particularly nitriding, influenced both surface roughness and wettability, with varying effects observed across different lubricants and droplet sizes. The findings provide valuable insights into the optimization of lubrication strategies for metal forming processes, highlighting the importance of tailored surface treatments for enhanced tool performance and longevity.

## 1. Introduction

The hot forging process is crucially influenced by the design of the tools and their working conditions. The accompanying high temperature and load set high demands on the tools used. The technological parameters affect the quality of the parts produced and the cost-effectiveness of the process. As in any manufacturing process, the aim is to achieve maximum efficiency, without replacing or repairing the tools [1,2,3,4,5,6,7]. The results of the research conducted and its analysis highlight the complexity of the process and the influence of parameters on the obtained results [1,4,8,9,10,11,12,13,14]. The selection of parameters and tool design should be analyzed in detail in each case [2,3,4,8,10]. Even a small change in values can significantly alter its course and the life of the tools [1,2,3,7,10]. It is especially important to analyze the lubrication process; adjusting it can significantly improve results [15,16,17,18].

The first important factor of hot forging is the material of the tools and their manufacturing method [1,2,3,4,5,17]. In the case of manufactured punches and dies, surface coatings are frequently used to increase the lifespan and improve the quality of the resulting forgings [4,5]. Research results confirm how the selection of the right material [2,3,17] or coating [4,5] can increase tool life. Punches during operation are most exposed to the occurrence of geometrical changes and surface damage due to the parameters of operation [1,2,4,5,12]. Lin et al. presented a study to test the feasibility of using a self-lubricating coating containing silver [5]. The results of the study confirmed that the use of such a coating helped to reduce tool wear when compared to a standard tool, and as a consequence, contributes to the increase in the number of forgings produced with a single tool and the extension in the tool’s useful life. A detailed analysis of the issue, together with the optimization of the die design, was shown to be successful in the reduction in wear in key areas of the punch [2].

The hot forging conditions have an influence on the final product and tool life. Besides the process parameters (temperature, contact time and pressure), the correct lubrication process of the tool is very important. The function of the lubricant during this manufacturing process is to reduce friction between the punch and the forging. The lubricant is also supposed to improve the thermal insulation between the components and protect the surface of the punch from wear while not causing additional damage to the punch itself [4,6,10,13,14,15,16,17,18,19,20]. The lubrication process should also ensure that the tool is cooled during operation but the intensity of the process should be controlled [6,15,19,20]. The operating parameters have an important influence on the selection of a suitable lubricant. In particular, the temperature, which can adversely affect the resulting lubricant’s coverage of the tool [19]. As with the choice of tool material, a detailed case study is required for the type of lubricant. There is a lack of universal solutions due to the influence of lubricant parameters [4,10,16,17] or method [18,20] on the results.

An important aspect is the agent application procedure. It should guarantee the formation of a proper layer of lubricant [11,14,18,19]. At the same time, cooling of the stamp is also important [6,15,19,20]. Consideration must be given to economic and environmental aspects [10].

Lubrication in hot forging is usually carried out by automatic or semi-automatic devices. Only when impossible or uneconomical is lubrication performed by manual spraying [15,20]. Automation of this process contributes to production cost reduction. Choosing the right lubrication parameters is especially important when the shape of the punch is irregular. The results of automation show that it is possible to improve hot forging conditions, leading to an increase in tool life and also allowing the quality and repeatability of the process to be increased [6,10,15,18,20,21]. Research by Wiadomski et al. presents the possibility of lubricating forged metal parts prior to the process, resulting in reduced decarburization of the surface layer [11].

Employing modern research methods and the analysis of results are expected to ensure better preparation of the process and the selection of optimum parameters [22]. Selecting the optimum values for the hot forging process parameters requires a great deal of experience and often experimental verification of the assumptions and results of the process simulations. Various factors influencing the results obtained have been studied [1,4,8,9,10,11,12,13,14]. A factor that affects the quality of lubrication is the medium used and the properties of the surface. How a liquid interacts with a solid or other liquid is defined by wettability. During the wettability test, the angle between the droplet and the solid surface is examined.

These are the following categories of surfaces according to the wetting angle θ:

- If θ < 50°, the surface is superhydrophilic;
- If 50° < θ < 90°, the surface is hydrophilic;
- If 90° < θ < 150°, the surface is hydrophobic;
- If θ > 150°, the surface is superhydrophobic [1].

**Figure 1:**
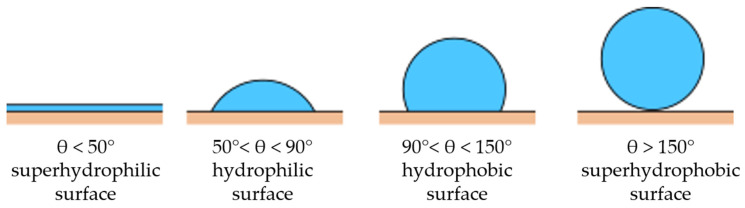
Demonstration of different surface wettabilities.

Examples of these angles and types of wetting are shown in Figure 1. The contact angle is mostly determined by the material properties of the wetted surface and its roughness, the characteristics of the wetting fluid and the droplet size [23,24,25,26].

This paper attempts to investigate the influence of a set of parameters on the wettability of hot forming punches. The influence of the punch surface finishing method, represented by surface topography parameters, was examined. Another factor tested was the type of liquid used and the droplet size. An analysis of the surface parameters for three treatments: after grinding, after polishing and after nitriding, is presented. The main part of the study was a wettability test carried out on a DataPhysics OCA15Pro (DataPhysics Instruments GmbH, Filderstadt, Germany) measuring machine. The results obtained and their analysis made it possible to determine the relevant relationships to help achieve the best possible production process.

## 2. Materials and Methods

**Figure 2:**
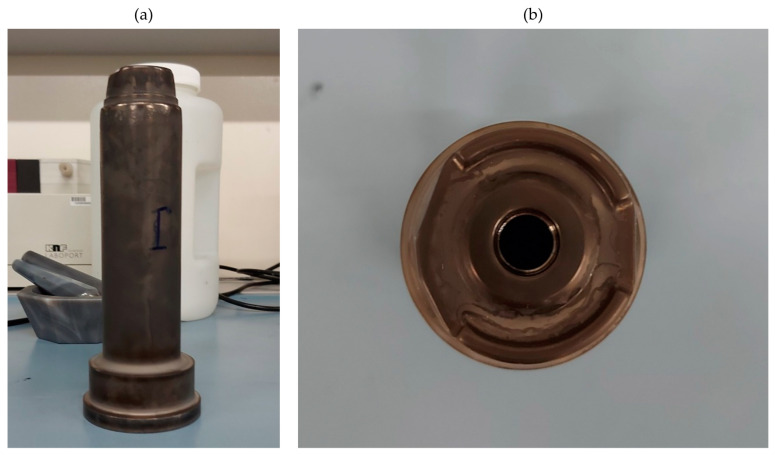
Picture of the stamp: (**a**) side view, (**b**) top view.

The subject of this study is the hot forging tool made of X40CrMoV5-1 (ISO 4957 [27]) hot work tool steel, as depicted in Figure 2. Two tools were manufactured and included in this study. They were tested in three different states of surface finish. For each condition, the machining parameters are given in Table 1, Table 2 and Table 3. The processing parameters presented in this study refer to the standard manufacturing process of the tool maker (Fabryka Armatur Swarzędz). These parameters were not intentionally optimized for enhanced wetting behavior. It was authors’ intention to conduct the study on a real manufacturing case study.

- As-ground.

**Table 1:** Grinding process information and parameters.

Parameter	Value
Machine	Jotes SPD 30c
Grinding wheel	Norton 350 × 50 × 127 mm 38 A 60 KVS
Grinding coolant	CIMTECH M21-02, concentration 5%
Depth of cut	0.02 mm
Grinding wheel speed	26 m/s
Grinding feed rate	15 mm
Table feed	20 m/min

- As-polished.

**Table 2:** Polishing process information and parameters.

Parameter	Value
Machine	Swepro SW 3371 M
Free speed of tool	60,000 1/min
Abrasives	Cratex Rubberized Abrasives with abrasive grains (silicon carbide) F version

- As-nitrided.

**Table 3:** Nitriding process information and parameters.

Parameter	Value
Type	Ion nitriding
Machine	JONIMP 900/500
Structure	Diffusion layer
Thickness of the diffusion layer	0.15–0.20 mm
Thickness of the nitride layer	4–5 µm
Hardness	900 HV
Temperature	540 °C
Pressure	5–10 hPa
Time	600 min
Atmosphere	N_2_ + H_2_

In this study, the wettability and surface topography of the tool were meticulously evaluated to understand the dependencies between surface characteristics and lubrication behavior. The research sought to determine whether and how the specific surface finishing influences the wettability properties when exposed to a lubricating fluid.

Wettability, defined by the contact angle between a liquid and a solid surface, plays a crucial role in the effectiveness of lubrication during hot forging processes. By analyzing the surface topography using advanced techniques (e.g., optical profilometry and microscopy), the study assessed roughness parameters induced by each surface processing. These data provide insights into how surface modifications, such as the nitriding layer applied to polished surfaces, affect lubricant distribution under high-temperature forging conditions. Understanding these relationships is essential for optimizing lubrication strategies and prolonging tool life in industrial forging processes.

**Figure 3:**
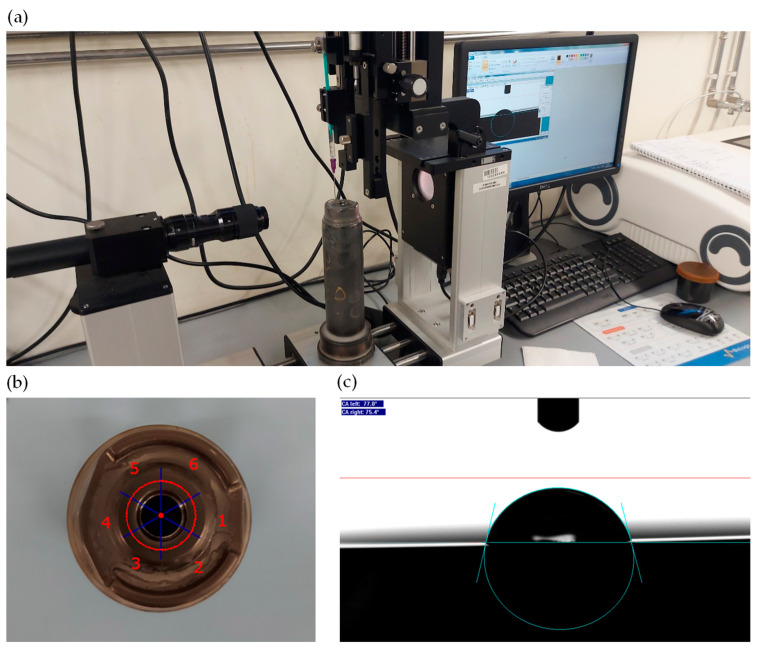
Wettability measuring: (**a**) measuring station, (**b**) indicative position of the measurement points, the points are located on the red line separated by blue lines, (**c**) example of wettability measurement.

The wettability tests were conducted using a DataPhysics OCA15Pro (DataPhysics Instruments GmbH, Filderstadt, Germany) measuring machine equipped with a goniometer, which consists of a camera, a liquid dispensing system, and a computer with SCA20 software (version 6.1, DataPhysics Instruments GmbH, Filderstadt, Germany) for OCA and PCA, used to control the camera and dispensing functions (Figure 3a).

The testing procedure began by placing the stamp on the goniometer. A syringe filled with one of the test fluids was then installed on the dispensing system. Three droplet volumes were tested:

- Volume of 0.5 µL with dosage 0.2 µL/s,
- Volume of 1 µL with dosage 0.5 µL/s,
- Volume of 2 µL with dosage 0.5 µL/s.

Each droplet was deposited at one of six pre-determined measurement points on the tool surface, as depicted in Figure 3b.

After dispensing, a 10 s wait allowed the droplet to stabilize on the surface before measuring the contact angle. Using the camera, the angle between the droplet and the surface was measured, with the system automatically identifying the edges of both. An example of this measurement setup is shown in Figure 3c.

Each measurement was repeated three times per point, across six points, on two different tools, in three surface finishes, with three different fluids, and at three dosages. This extensive setup resulted in a total of 972 individual measurements. As each measurement yielded two angle values (representing both sides of the droplet), the study accumulated 1944 data points for comprehensive analysis.

The surface topography analysis began with positioning the tool under an Alicona PortableRL (Alicona Imaging GmbH, Raaba, Austria) focal differentiation microscope, capable of reconstructing surface topography from a series of 2D images captured between the lowest and highest focal points. This setup enabled a detailed 3D scan of the tool surface, capturing topographical information across the studied area. In this study, we used 20× magnification lens and studied an area of 1 mm by 1 mm (in x- and *y*-axis). Measured datasets were then postprocessed using MountainsMap software (version 10.2, Digital Surf, Besançon, France). Firstly, measurements were leveled and then form was removed. Surface roughness was extracted using Gaussian filter with cut-off value equal to 0.8 mm. A number of areal surface parameters were calculated for each test specimen (see Table A1, in Appendix A). The distribution of the parameters is presented using box-and-whisker charts.

Following scanning, the raw data were analyzed in Mountains software (version 10.2, Digital Surf, Besançon, France), which constructed a three dimensional model of the surface.

Finally, a statistical analysis of the calculated results was performed using Wolfram Mathematica (version 14.0, Wolfram Research, Champaign, IL, USA) and MS Excel (version 16, Microsoft, Redmond, WA, USA). To test the effect of finishing state, wetting fluid and droplet size, three-way ANOVA was conducted. Significance level was assumed as 0.05. Relations between contact angle and surface areal parameters were evaluated using coefficient of determination (R^2^).

## 3. Results

### 3.1. Surface Measurements

**Figure 4:**
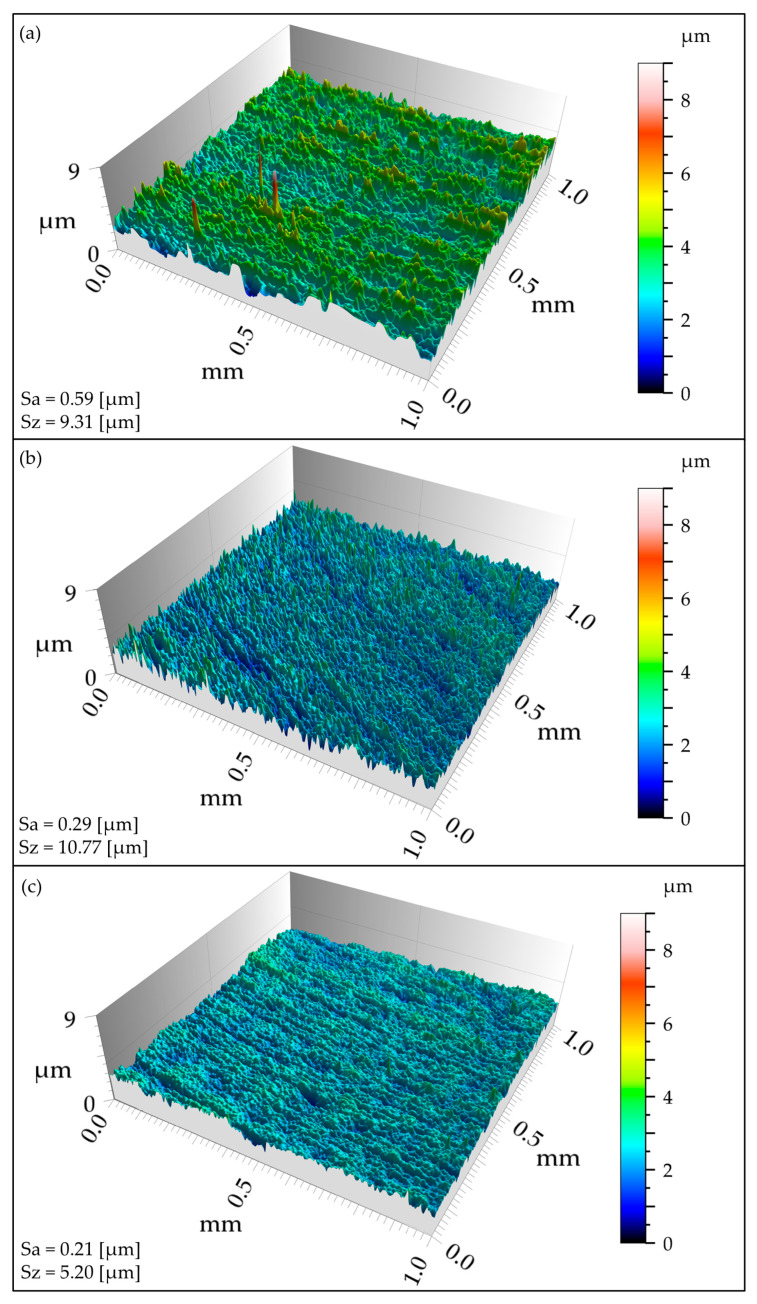
Example of surface measurement result, (**a**) after grinding, (**b**) after polishing, (**c**) after nitriding of previously polished surfaces.

Color-coded height maps of the representative examples of different surface finished topographies are shown in Figure 4. It should be noted that all analyzed surfaces show a similar directional morphology with a decreasing amplitude of the heights. As expected, surface polishing contributes to a significant decrease in the heights of the asperities. The visible anisotropy is caused by the directional interaction with the grinding tool and polishing compounds in motion. The nitriding does not influence the surface morphology but only results in the drop in surface roughness.

**Figure 5:**
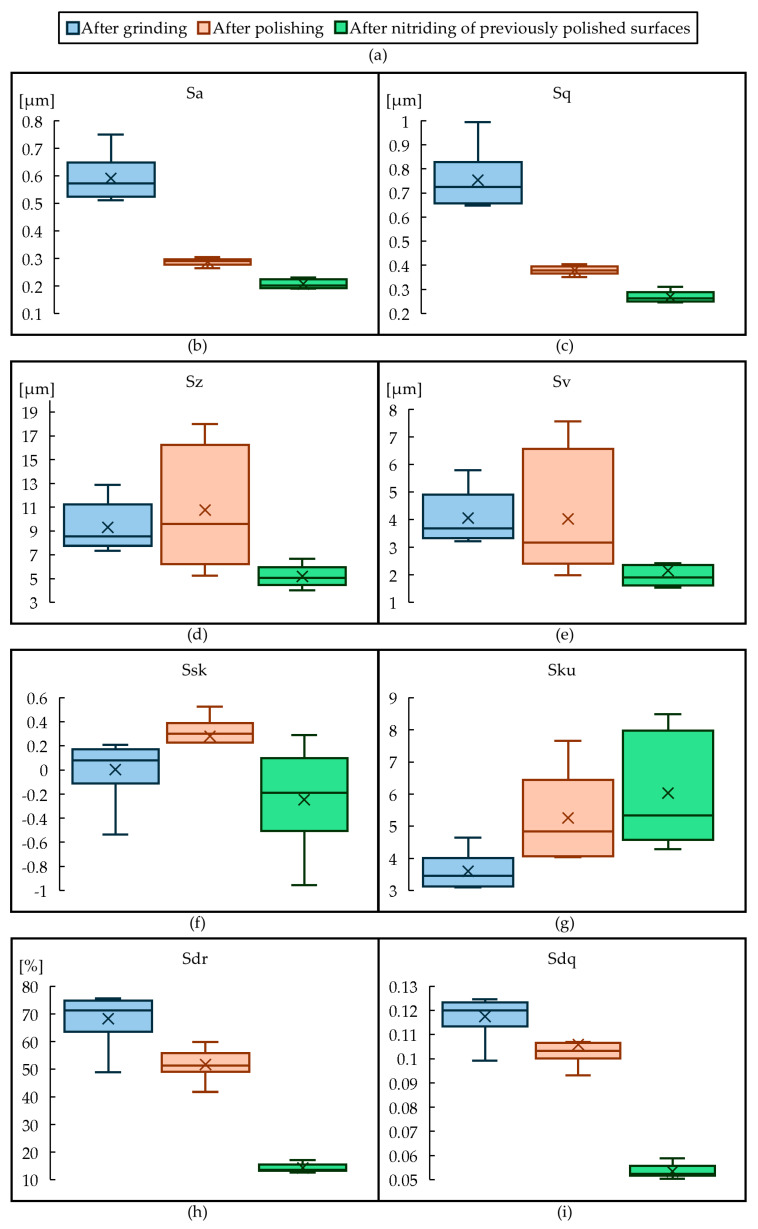
Box and whisker plots for surface parameters of the stamps obtained after the three finishing methods; the legend to (**b**–**i**) can be found in (**a**).

Figure 5 shows the distribution of the Sz parameter for different surface finishes. With each successive processing phase, Sz becomes reduced. Polishing creates a large dispersion of Sz when compared to as-ground and as-nitrided. This can be explained by the fact that polishing is a manual process and its repeatability compared to grinding is lower. Nitriding mitigates this phenomenon understood as a reduction in the Sz variation. With each successive processing phase, the kurtosis of the height distribution (Sku) increases. The height distribution is therefore more and more concentrated, meaning that the surface become less rough. Ssk changes from 0 to positive and negative values. This shows a transformation between peak-dominant to valley-dominant topographies. With each successive finishing state, Sdr values become smaller, which means that the geometric complexity of the surface decreases. The same observation can be made for Sdq, which means that the average slope of the surface asperities is reduced.

**Figure 6:**
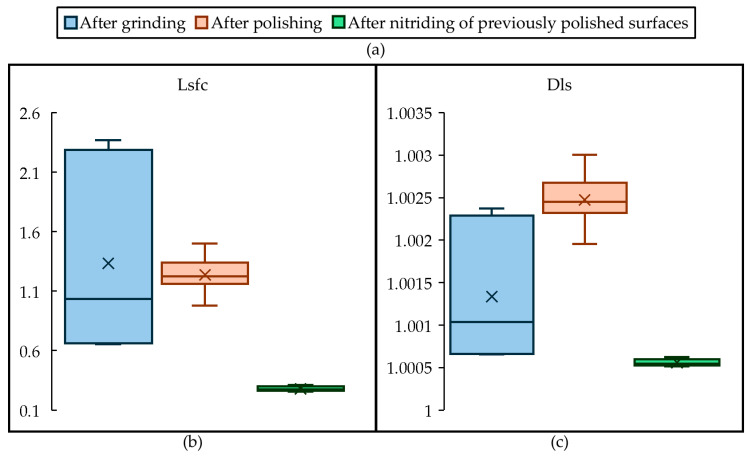
Box-and-whisker plots for surface parameters of the stamps obtained after the three finishing methods, the legend to (**b**,**c**) can be found in (**a**).

The Lsfc and Dls parameters are scale-dependent fractal analysis parameters, defining the relevant fractal complexities and fractal dimensions, respectively. The graph in Figure 6 shows the distributions of the Lsfc and DIs parameters as a function of the processing state. With successive machining phases, Lsfc decreases and DIs increases. The distribution of both also narrows. This observation shows that polishing and nitriding significantly reduce the fractal complexity of surface topographies.

### 3.2. Wetting Angle Values

**Figure 7:**
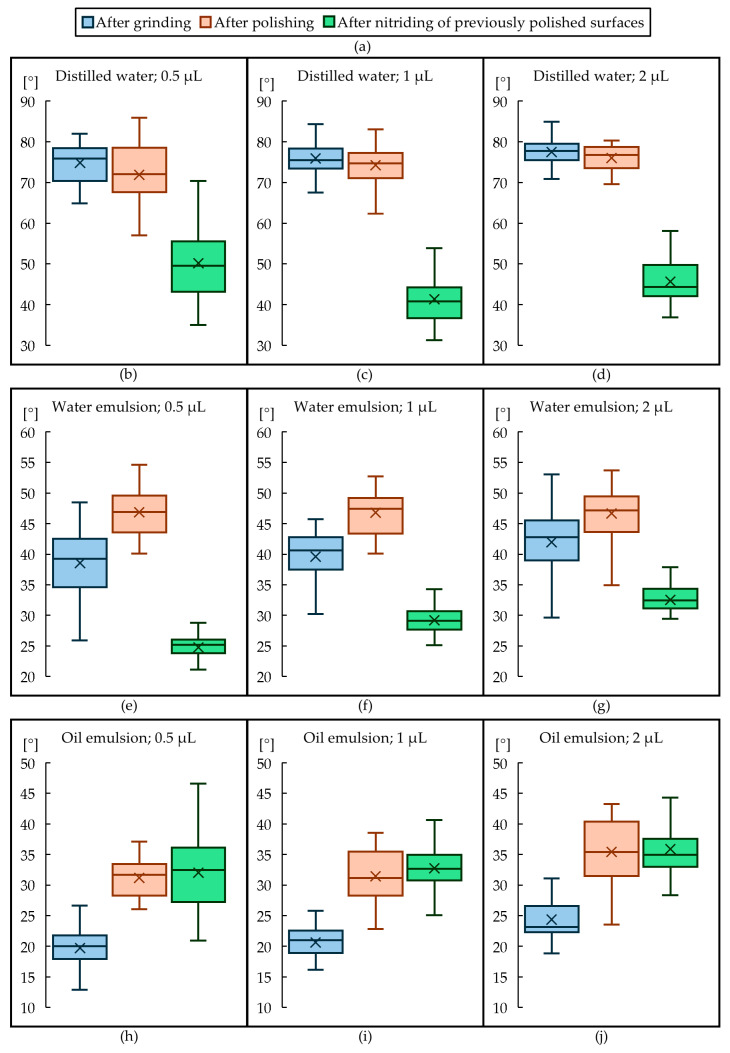
Box-and-whisker plots showing the obtained values of wetting angles for the three liquids tested, the three droplet sizes and for the different stamp finishing treatments, the legend for plots (**b**–**j**) can be found in (**a**).

The distributions of the measured contact angles for the different droplet sizes, type of liquid and the surface finish are presented using box-and-whisker plots as shown in Figure 7.

The plots presented in Figure 7 shows that water-based liquids have a greater wetting angle than oil-based ones. Regardless of the treatment, distilled water has the largest wetting angle, water emulsion has a smaller one and oil emulsion has the smallest wetting angle. This can be explained by the viscosity of the fluid which contributes to the increased contact angles.

### 3.3. Correlation Between Surface and Wetting Parameters

ANOVA was performed to determine the statistical significance between surface finish and wetting parameters. It was shown that all analyzed surface areal parameters were statistically significant (*p* < 0.001) in telling the surfaces apart. The results of this analysis for the results shown in Figure 5 are shown in Table 4.

Using three-way ANOVA, we show that all factors: droplet size, finishing method and fluid type, have a significant effect on the contact angle (*p* < 0.001); results of the analysis are presented in Table 5. This effect is strong if those factors are considered individually or as a combination.

To determine the impact of individual surface parameters on the wetting angle for various wetting liquids, a linear regression analysis was conducted. Table 6 presents these results. If the coefficient of determination (R^2^) was greater than 0.5, it was assumed that the given parameter affected wettability. It was noted that the parameters Sa and Sq enhanced wettability (decreased the wetting angle) only for the oil emulsion. However, these parameters were found not to correlate with contact angles for the other liquids. An increase in the parameters Sz, Sv, and Ssk reduced wettability (increased the wetting angle) for distilled water and the water emulsion. This effect was not observed for the oil emulsion.

An increase in the Sku parameter enhances wettability for distilled water but reduces wettability for the oil emulsion. For the water emulsion, the Sku parameter has no impact on wettability. It can also be observed that an increase in the Sdr parameter reduces wettability with distilled water but positively impacts wettability with both the water and oil emulsions. In addition, an increase in the Sdq and Lsfc parameters reduces wettability with distilled water and the water emulsion. These parameters have no effect on wettability with the oil emulsion.

## 4. Discussion

The study systematically evaluated the influence of surface finish, heat treatment, and fluid characteristics on the wettability of hot-forming tools. Wettability, quantified through contact angle measurements, was assessed for three surface states (as-ground, as-polished, and as-nitrided) and correlated with surface topography and fluid properties. Surface topography analysis revealed that polishing reduced asperity heights, while nitriding further decreased surface roughness and improved repeatability. Fractal analysis demonstrated a reduction in fractal complexity with successive treatments. Wettability tests showed that water-based fluids exhibited larger contact angles than oil-based emulsions, influenced by fluid viscosity and surface characteristics. Statistical analysis (three-way ANOVA) confirmed significant effects of surface finish, fluid type, and droplet size on contact angles. Regression analysis highlighted specific topographical parameters (e.g., Sa, Sz, Sku) as key influencers of wettability, with varying impacts across fluid types. These findings underscore the importance of surface engineering in optimizing lubrication performance and tool durability in hot forging applications.

The specific geometric measures of the surface texture and its individual features, such as the height, diameter, and spacing of microstructures, play a crucial role in determining the contact angle. For instance, increasing the height of micro-textures generally increases the contact angle, enhancing hydrophobicity [28]. The Cassie–Baxter model involves the parameter which corresponds to Sdr (according to ISO 25178 [29]). The higher the Sdr, the higher the contact angle [23]. In our work, we demonstrated that decreasing the surface roughness with subsequent surface processing leads to a drop in contact angle. Further nitriding promoted the hydrophilicity. This is in contrast to the study by Borgioli et al. [30], in which the authors found that nitriding contributed to the increased surface roughness, which played a crucial role in altering the wetting properties. However, the authors used low temperature nitriding and mirror polished substrate surfaces, which, unlike our observations, resulted in the formation of the new topographic features, i.e., peaks and valleys of higher amplitude. The rougher surfaces created by nitriding can lead to higher apparent contact angles. In our observation, nitriding decreased the surface roughness (as described by Sa, Sz or Sdr), which had a major impact on the resulting reduction in contact angle. Some examples showed that nitride steels exhibited low contact angles when soldered, but no reference to untreated surfaces as a control group was given [31,32]. In carburization, which shows some similarity to nitriding, the decrease in contact angle is noted. This happens due to the altering of surface chemistry and promoting the formation of carbides [33,34,35].

A key finding, unique to this study, is the consistent relationship between reduced roughness and enhanced wetting for water-based lubricants, which contrasts with the behavior observed for oil-based emulsion. While water-based fluids exhibited larger wetting angles on rougher surfaces, the hydrophilicity improved dramatically on smoother, nitrided surfaces. This behavior can be attributed to the surface’s interaction with the fluid’s viscosity and surface energy, as evident in our linear regression analysis. Nitriding not only altered the surface topography but also introduced a chemically modified nitride layer. This layer likely increased the surface energy, facilitating better interaction with polar fluids like water-based lubricants. Regression analysis confirmed that specific surface parameters (e.g., Sdr and Sdq) were inversely correlated with contact angle, underscoring the synergistic effects of topographical and chemical changes.

The effect of droplet size on the contact angle is a well-understood phenomenon [36]. In the microliter range, as studied here, Drelich [37] concluded that droplet volume has no significant influence on the contact angle with close-to-ideal surfaces, such as clean quartz plates. Yang et al. [38] compared pico- and microliter droplet water contact angles on grooved polymethyl methacrylate (PMMA) surfaces coated with plasma polymers as a first study to investigate anisotropic wetting behavior with picoliter droplets. They found significant differences in water contact angles when varying the contact angle from microliters to picoliters, and therefore highlighted the importance of showing drop size alongside contact angle results. Vafei and Podowski reported that the contact angle decreases with an increase in the size of a droplet [39] for rough surfaces. The same observations were noted in our work for water and oil emulsion. The differences for distilled water were more subtle in values.

This study’s systematic evaluation of fractal parameters also provides new insights into the role of surface complexity in wettability. The observed reduction in fractal complexity (e.g., lower Lsfc and Dls values) with successive treatments highlights how machining and nitriding techniques can influence surface topography, making it more amenable to uniform lubricant spreading. These insights are particularly relevant for optimizing lubrication strategies in industrial settings where uniform fluid distribution is critical.

By correlating these findings directly with the materials and methods employed, this study offers practical guidance for tool preparation in hot forging processes. For instance, the observed benefits of nitriding to improve lubricant coverage and reducing wear suggest that tailored surface treatments can enhance tool performance and longevity. Unlike generalized observations from prior research, the current work provides actionable recommendations for the specific conditions encountered in high-temperature forging with X40CrMoV5-1 steel.

The scientific merit of this paper lies in its comprehensive integration of surface topography analysis with wettability testing under realistic wetting conditions in forging. By directly linking specific surface modifications to practical lubrication outcomes, this study bridges a critical gap between experimental observations and industrial applications. Moreover, the novel exploration of fractal parameters in this context provides a deeper understanding of surface behavior, setting a foundation for future studies in this field.

Future work should extend our observations by investigating the long-term effects of wear on wettability and exploring how tool regeneration impacts surface properties. Such studies could further refine the recommendations for maintaining optimal lubrication behavior over the tool’s lifecycle.

## 5. Conclusions

This study provides a detailed investigation into the influence of surface finishing, lubrication characteristics, and other operational parameters on the wettability of hot forming punches, with a focus on optimizing tool performance and lubrication strategies in the hot forging process. The findings highlight the significant role of surface treatments, lubricating fluids, and droplet size in determining the contact angle, which directly influences the effectiveness of lubrication and, consequently, tool life and product quality.

The analysis of surface topography revealed that different surface finishes (as-ground, as-polished, and as-nitrided) considerably impacted the wettability of the tool surfaces. The nitrided surfaces, in particular, demonstrated more favorable wetting properties compared to the as-ground and as-polished finishes, indicating that nitriding not only enhances surface hardness but also improves lubrication behavior by fostering better fluid distribution. This mechanism can be explained by lowering of the surface roughness. This result underscores the importance of surface modifications in optimizing lubricant coverage and reducing tool wear during the high-temperature forging process.

In addition, the study confirmed that the type of lubricating fluid and the size of the droplets significantly influenced the wettability measurements. Smaller droplet sizes and specific lubricants exhibited improved spreading and contact angle reduction, which enhances overall lubrication efficiency during forging operations. This suggests that the careful selection of lubricants, in combination with optimized droplet dispensing techniques, can contribute to better tool performance and extended tool life.

The results presented in this paper encourage further research in this area. A next part of the study should be to examine the effect of tool wear and tool regeneration on the wettability. By doing so, it would be possible to select the most suitable regeneration method for the tool and to analyze the effect of wear on wettability.

## Figures and Tables

**Table 4:** Results of the ANOVA analysis presenting surface finishing-treatment effect on surface parameters.

Parameter	*p*-Value
Sa	<<0.001
Sq	<<0.001
Sz	8.56 · 10^−3^
Sv	3.86 · 10^−2^
Ssk	1.35 · 10^−2^
Sku	1.43 · 10^−2^
Sdr	<<0.001
Sdq	<<0.001

**Table 5:** Results of the three-way ANOVA analysis presenting droplet size, surface finishing-treatment and liquid-type effect on wetting angles.

Factor	*p*-Value
Droplet size	<<0.001
Surface finishing treatment	<<0.001
Liquid type	<<0.001
Droplet size and surface finishing treatment	<<0.001
Droplet size and liquid type	<<0.001
Surface finishing treatment and liquid type	<<0.001
Droplet size x surface finishing treatment and liquid type	<<0.001

**Table 6:** Coefficients of determination (R^2^) of the impact of individual surface parameters on the wetting angle for various wetting liquids; I—increase in the wetting angle; D—decrease in the wetting angle;—has no impact.

Liquid Type	Droplet Size	Sa		Sq		Sz		Sv		Ssk		Sku		Sdr		Sdq		Lsfc	
Distilled water	0.5	0.552	I	0.565	I	0.873	I	0.825	I	0.621	I	0.664	D	0.963	I	0.997	I	0.994	I
	1	0.487	I	0.500	I	0.913	I	0.879	I	0.683	I	0.601	D	0.934	I	0.985	I	0.998	I
	2	0.485	I	0.498	I	0.914	I	0.882	I	0.685	I	0.599	D	0.933	I	0.984	I	0.998	I
Water emulsion	0.5	0.116	–	0.156	–	0.985	I	0.967	I	0.970	I	0.199	–	0.601	D	0.729	I	0.799	I
	1	0.093	–	0.101	–	0.974	I	0.963	I	0.982	I	0.170	–	0.563	D	0.694	I	0.168	I
	2	0.147	–	0.012	–	0.994	I	0.974	I	0.953	I	0.237	–	0.644	D	0.768	I	0.834	I
Oil emulsion	0.5	0.980	D	0.976	D	0.105	–	0.128	–	0.001	–	0.935	I	0.603	D	0.442	D	0.385	D
	1	0.989	D	0.986	D	0.129	–	0.134	–	0.005	–	0.952	I	0.640	D	0.505	D	0.422	D
	2	0.972	D	0.967	D	0.090	–	0.115	–	0.000	–	0.922	I	0.578	D	0.468	D	0.360	D

## Data Availability

The original contributions presented in this study are included in the article. Further inquiries can be directed to the corresponding author.

## Appendix A

**Table A1:** Calculated areal surface parameters [29].

Sa	
$S_{a}=\frac{1}{A}\iint_{A}\left|z\left(x,y\right)\right|dxdy\left[\mu m\right],\;(1)$ where A—scanned area, z—surface height, x—axis coordinate x, y—axis coordinate y	The parameter Sa is, according to ISO 25178, the arithmetic mean height of the area. This parameter is commonly used to assess the roughness of a surface
Sq	
$S_{q}=\sqrt{\frac{1}{A}\iint_{A}z^{2}\left(x,y\right)dxdy}\left[\mu m\right],\;\left(2\right)$ where A—scanned area, z—surface height, x—axis coordinate x, y—axis coordinate y	The parameter Sq is, according to ISO 25178, the root mean square of area height. It is equivalent to the standard deviation of heights
Sz	
Sz=z(x,y)x,y∈Amax−zx,yx,y∈Aminμm, (3) where A—scanned area, z—surface height, x—axis coordinate x, y—axis coordinate y	The parameter Sz is, according to ISO 25178, the sum of the largest peak height value and the largest pit depth value within the specified area
Sv	
$S_{v}=\left|\underset{A}{\min}z(x,y)\right|\left[\mu m\right],(4)$ where A—scanned area, z—surface height, x—axis coordinate x, y—axis coordinate y	The parameter Sv is, according to ISO 25178, the absolute value of the height of the largest pit within the specified area
Ssk	
$S_{sk}=\frac{1}{{S_{q}}^{3}}\left[\frac{1}{A}\iint_{A}Z^{3}\left(x,y\right)dxdy\right]\left[\mu m\right],(5)$ where Sq—root mean square of area height A—scanned area, z—surface height, x—axis coordinate x, y—axis coordinate y	The parameter Ssk is, according to ISO 25178, the value that represents the degree of bias of the roughness shape (asperity). If Ssk < 0, height distribution is skewed above the mean plane, if Ssk = 0, height distribution (peaks and pits) is symmetrical around the mean plane and if Ssk > 0, height distribution is skewed below the mean plane
Sku	
$S_{ku}=\frac{1}{S_{q}^{4}}\frac{1}{A}\iint_{A}z^{4}(x,y)dxdy\left[\mu m\right],(6)$ where Sq—root mean square of area height A—scanned area, z—surface height, x—axis coordinate x, y—axis coordinate y	The Sku parameter is, according to ISO 25178, the kurtosis of the height distribution.This parameter is used to evaluate sharpness in the height distribution. If Sku < 3, height distribution is even, if Sku = 3, height distribution is normal (sharp portions and indented portions co-exist) and if Sku > 3, height distribution is sharp
Sdr	
$S_{dr}=\frac{1}{A}\left[\iint_{A}\left(\sqrt{\left[1+{\left(\frac{\partial z\left(x,y\right)}{\partial x}\right)}^{2}+{\left(\frac{\partial z\left(x,y\right)}{\partial y}\right)}^{2}\right]}-1\right)dxdy\right]$, (7) where A—scanned area, z—surface height, x—axis coordinate x, y—axis coordinate y	The Sdr parameter, according to ISO 25178, indicates the rate of growth in the superficial area. The Sdr of a perfectly flat surface is 0. As the surface gains any degree of slope, its Sdr value increases. Sdr is the hybrid parameter
Sdq	
$S_{dq}=\sqrt{\frac{1}{A}\iint_{A}\left[{\left(\frac{\partial z(x,y)}{\partial x}\right)}^{2}+{\left(\frac{\partial z(x,y)}{\partial y}\right)}^{2}\right]dxdy}$, (8) where A—scanned area, z—surface height, x—axis coordinate x, y—axis coordinate y	The Sdq parameter is, according to ISO 25178, the root mean square area gradient. This parameter represents the average size of the surface’s local slopes. A perfectly horizontal surface has Sdq = 0. A higher Sdq value means the surface has steeper inclines. Sdq is the hybrid parameter
Dls and Lsfc	
Lsfc and DIs are fractal analysis parameters. Dls is fractal dimension while the Lsfc (length-scale fractal complexity) characterizes how much the profile is more complex than a Euclidian line	
